# Enhanced tuberculosis case detection among substitution treatment patients: a randomized controlled trial

**DOI:** 10.1186/1756-0500-4-192

**Published:** 2011-06-15

**Authors:** Kristi Rüütel, Helle-Mai Loit, Tiiu Sepp, Kai Kliiman, Louise-Anne McNutt, Anneli Uusküla

**Affiliations:** 1 Department of Infectious Diseases and Drug Abuse Prevention, National Institute for Health Development, Tallinn 11619, Estonia; 2 Department of Chronic Diseases, National Institute for Health Development, Tallinn 11619, Estonia; 3 LLC Corrigo, Jõhvi 41532, Estonia; 4 Lung Clinic, Tartu University Hospital, Tartu 51014, Estonia; 5 School of Public Health, University at Albany, State University of New York, Rensselaer, NY 12144, USA; 6 Department of Public Health, University of Tartu, Tartu 50411, Estonia

## Abstract

**Background:**

Historically, HIV, TB (tuberculosis) and illegal drug treatment services in Estonia have been developed as vertical structures. Related health care services are often provided by different health care institutions and in different locations. This may present obstacles for vulnerable groups, such as injecting drug users (IDU), to access the needed services. We conducted a small scale randomized controlled trial to evaluate a case management intervention aimed at increasing TB screening and treatment entry among IDUs referred from a methadone drug treatment program in Jõhvi, North-Eastern Estonia.

**Findings:**

Of the 189 potential subjects, 112 (59%) participated. HIV prevalence was 86% (n = 96) and 7.4% (n = 8) of participants were interferon gamma release assay (IGRA) positive (6.5% were both HIV and IGRA-positive, n = 7). Overall, 44% of participants (n = 49) attended TB clinic, 17 (30%) from control group and 32 (57%) from case management group (p = 0.004). None of the participants were diagnosed with TB. In a multivariate model, those randomized to case management group were more likely to access TB screening services.

**Conclusions:**

These findings demonstrate the urgent need for scaling up TB screening among IDUs and the value of more active approach in referring substitution treatment patients to TB services.

**Trial registration:**

ClinicalTrials.gov: NCT01290081

## Background

Illegal drug use has become a risk factor for tuberculosis (TB) as a result of the overlap of epidemiological and social factors associated with both drug use and TB. The spread of HIV infection has further amplified the spread of TB among drug users [1,2]. An increase of tuberculosis among relatively young individuals such as injecting drug users (IDU) who often have active social lives may have serious public health consequences and lead to the spread of tuberculosis infection in the environment. Therefore, HIV infected persons and persons at risk for HIV infection should be closely monitored for TB even in a country where the prevalence of tuberculosis is low [3].

Estonia, located in North-Eastern Europe, is the smallest of the Baltic nations with a total population of approximately 1.3 million people, had the TB incidence rate of 26.2/100,000 in 2008; the proportion of multi-drug resistant (MDR) cases (defined as TB caused by *M. tuberculosis *resistant *in vitro *to isoniazid and rifampicin) among new TB cases was 11.9% and among relapses 29.5% respectively. The proportion of TB patients co-infected with HIV was 9.4% [4,5]. Estonia also has the highest numbers of newly diagnosed HIV cases per 100,000 population in the European Union [6]. The adult injecting drug use prevalence (among 15-44 year old) is estimated to be 2.4% [7] and HIV-prevalence rates as high as 70% have been described among IDUs [8]. Previous research has shown that up to 2% of IDUs have been diagnosed with TB in lifetime. In neighboring country Latvia this percentage is even higher - 7% [9].

Historically, HIV, TB and illegal drug treatment services in Estonia have been developed as vertical structures. Related health care services are often provided by different health care institutions and in different locations. This may present obstacles for vulnerable groups, such as injecting drug users, to access the needed services. Directly observed treatment short course (DOTS) has been implemented in Estonia since 2000 and DOTS plus strategy for MDR-TB treatment has been implemented since August 2001. The DOTS and DOTS plus coverage is 100%. Health care services related to TB diagnostics and treatment are free of charge for all patients [4].

International guidance often supports the idea of one-stop shop or integration and co-location of HIV, tuberculosis and drug treatment services. While the fully integrated approach should be viewed as the ideal situation, it may not be attainable immediately due to funding shortfalls or organizational and political constraints. As such, it should be viewed as a goal to be evolved over time [10]. In situations where co-locating services is not possible, other measures can be implemented to increase uptake and coverage of services. Most crucial is efficient collaboration between different service providers. In programs that use additional outreach techniques, such as assistance with transportation, incentives, and food vouchers, very high levels of participation and adherence have been achieved in populations facing the most difficult barriers, such as homelessness, severe mental illness, and active substance abuse [11-13].

The current project sought to test alternative methods in referral of substitution treatment patients to TB control services in order to develop a model of collaboration for TB and drug treatment services.

## Methods

We conducted a small scale randomized controlled trial to evaluate a case management intervention aimed at increasing tuberculosis screening and treatment entry among injecting drug users referred from a methadone drug treatment program in Jõhvi, North-Eastern Estonia.

### Setting and sample

Subjects were recruited from a community-based methadone substitution treatment center in Jõhvi. The center has 250 slots for treatment and operates seven days a week. It is funded by the government and services are free of charge for the patients.

Initial recruitment took place during regular business hours of the treatment center in October 16-18, 2007. All consecutive clients were approached for the study participation.

Recruitment and study randomization was executed by medical nurses who were full-time employers of the substitutions treatment center. They received in-house training regarding standardization of study protocol, procedures to ensure confidentiality, and Mantoux testing. They had been trained also on HIV counseling and testing.

Eligibility criteria included participation in substitution treatment program, age 18 years or more, able to read and write in Estonian or Russian, and able to provide informed consent. In order to link the data from different study sites (substitution treatment center and TB services) each participant was given a unique code.

Consenting participants were referred for TB screening (chest X-ray and additional tests if necessary) to the tuberculosis department of the local county hospital (distance between the two centers is approximately 16 km).

### Study instruments and procedures

1. At the substitution treatment center:

1) Socio-demographic data, TB and IDU history, imprisonment, and TB contacts were collected using a semi-structured questionnaire which was modified from multiple questionnaires [14-17]. The questionnaire was designed for self-administration and required approximately 20 minutes to complete.

2) Whole venous blood was drawn for HIV and interferon-γ testing. Pre- and post-test counseling was provided by study nurses. HIV test results were available at the follow-up visit to Mantoux test reading and interferon-γ test results after three weeks of initial participation.

3) HIV tests were performed at National HIV Reference Laboratory (using Vironostika HIV Uniform II Ag/Ab; BioMerieux; positive cases were confirmed with INNO LIA HIV I/II Score Westernblot).

4) Interferon-γ testing was performed at National Tuberculosis Reference Laboratory using an IGRA assay (QuantiFERON-TB Gold; Cellestis Europe^©^). The technology is based on the measurement of interferon-gamma (IFN-) secreted from stimulated T-cells previously exposed to *Mycobacterium tuberculosis *[18]. CDC recommends interferon-gamma release assays (IGRA) in place of TB skin testing in all situations where tuberculin skin testing is recommended as an aid in diagnosing *M. tuberculosis *infection. An IGRA is preferred for testing persons who have received BCG vaccination [19]. In Estonia, BCG vaccination is part of the recommended immunization schedule and is performed to all newborns by the fifth day after birth [20].

5) Mantoux skin testing using 2TU of PPD 23 SSI. Test results were read after 48-72 hours. Induration of ≥5 mm was considered as positive result.

6) Participants who returned to skin test reading were randomly assigned by the study nurses into (1) passive referral group - control group (were instructed to schedule an appointment with TB services themselves); or (2) active referral group - case management intervention group (study personnel scheduled the appointment and reminded to keep it, transportation was organized when needed). Participants were expected to attend TB services within the two months after the initial randomization. For those who returned to skin test reading on time an incentive was given (food voucher for local supermarket with the value of 6.4 EUR).

2. At the tuberculosis department:

1) Participants who attended TB services were screened for active tuberculosis according to the clinical guidelines of Estonian Respiratory Society [21].

2) Medical doctors filled in a semi-structured questionnaire with the main demographic characteristics, additional tests and procedures done, and the final diagnosis of the participant based on ICD-10.

### Statistical analysis

The interviewers filled questionnaires during the interviews, and checked them immediately after the interview. Completed questionnaires were collected, checked and reviewed for inaccuracies by supervisors on a daily basis. All data was entered twice and the data sets were compared to detect and correct any data entry errors. Statistical analyses were performed with STATA 11.0 (StataCorp LP. College Station, TX). Descriptive statistics were used to characterize participants by intervention group and attendance to TB services. Associations between participant characteristics and attendance to TB services were evaluated by using the Wilcoxon rank-sum test or Fisher exact test, followed by univariate and multivariable logistic regressions.

### Ethical committee

The study was approved by the Tallinn Medical Research Ethics Committee and all participants provided oral informed consent.

## Results

### Population characteristics

A total of 189 people were invited to participate in the project. The study included 112 respondents (participation rate 59%). The reasons for refusals were recent visit to TB doctor (n = 34; 46%), no time to participate (n = 22; 29%), did not want to fill in the questionnaire (n = 17; 25%). Participants and refusals did not differ in mean age, and gender. Timely return rate for skin test reading was 100% and thus all people initially participating were randomized.

86% of participants (n = 96) were HIV-positive and 7.4% were IGRA-positive (n = 8). Among HIV-positive participants 6.5% were IGRA-positive (n = 6) and among HIV-negative participants - 14.3% (n = 2)(p = 0.3). Among IGRA-negative participants 13.0% (n = 14) and among IGRA-positive - 62.5% were Mantoux-positive (n = 5)(p = 0.003).

None of the participants reported ever having had TB. 35 participants (31%) had had chest X-ray done in last year before the study. 27 (24%) had the last X-ray more than three years before the study and 12 (11%) did not remember the time of the last X-ray.

Detailed socio-demographic characteristics, TB and drug use history, and test results of the participants in control and case-management groups are presented in Table 1.

**Table 1:** Socio-demographic characteristics, TB and drug use history, and test results of the participants

Characteristic	Total	Control group	Case management group
Male participants (%, 95% CI)	64.9 (55.8-73.9)	61.8 (48.7-74.9)	67.9 (55.4-80.3)
Mean age in years (SD; range)	26.2 (4.2; 19-48)	26.3 (3.1; 20-33)	26.0 (5.2; 19-48)
Sample younger than 30 years of age (%, 95% CI)	83.9 (77.0-90.8)	82.1 (71.9-92.4)	85.7 (76.4-95.1)
Ethnicity (%, 95% CI)			
Estonian	9.8 (4.2-15.4)	8.9 (1.3-16.5)	10.7 (2.5-19.0)
Other	90.2 (84.6-96.8)	91.1 (83.5-98.7)	89.3 (81.0-97.5)
Persons with less than 12 years of education (%, 95% CI)	56.3 (46.9-65.6)	51.8 (38.4-65.1)	60.7 (47.7-73.8)
Percentage of employed participants (%, 95% CI)	49.1 (39.7-58.5)	48.2 (34.9-61.6)	50.0 (36.6-63.4)
Percentage of participants who had been in prison at least once in lifetime (%, 95% CI)	61.6 (52.5-70.6)	67.9 (55.4-80.3)	55.4 (42.1-68.6)
Mean age at initiation of IDU (SD; range)	16.8 (3.3; 11-30)	17.3 (3.6; 11-26)	16.2 (3.0; 11-30)
Mean duration of IDU in years (SD; range)	9.4 (3.6; 2-24)	9.0 (3.4; 2-20)	9.8 (3.8; 4-24)
Percentage uninsured by the national health insurance (%, 95% CI)	36.6 (27.5-45.7)	35.7 (22.9-48.5)	37.5 (24.6-50.4)
Percentage of people with TB contacts (%, 95% CI)	24.1 (16.1-32.2)	66.7 (53.4-79.9)	81.1 (70.4-91.9)
Percentage of people who had had TB (%, 95% CI)	0.0	0.0	0.0
Percentage HIV-antibody positive	86.2 (79.7-92.8)	88.7 (80.0-97.4)	83.9 (74.1-93.7)
Percentage IGRA-positive (%, 95% CI)	7.4 (2.4-12.4)	9.4 (1.4-17.5)	5.5 (0.0-11.6)
Percentage Mantoux-positive (≥5 mm) (%, 95% CI)	17.0 (9.9-24.0)	19.6 (9.0-30.3)	14.3 (4.9-23.6)

### Attendance to TB services

By design, 56 (50%) were randomized to the intervention and 56 (50%) to the control condition. There were no baseline differences between the intervention and control groups with respect to any socio-demographic or behavioral characteristics, duration of injecting drug use, previous exposure to TB or ever having had TB.

43.8% of participants (49/112) attended TB clinic, 17 (30.4%) from control group and 32 (57.1%) from case management group (p = 0.004). None of them were diagnosed with TB.

We examined the relationships between several potential predictive variables assessed at intake and the outcome measure - attendance to TB services. Potential predictors included age older than 29 years, female gender, more than nine years of education, being employed, injecting drugs less than 10 years, ever been in prison, previous TB contacts, Mantoux and HIV test results, and type of referral (active versus passive). Both in simple and multivariable logistic regression analysis none of the candidate variables was significantly associated to attendance to TB services, except for the type of referral. Thus people from case management group had 3.9 (95% CI 1.4-10.4) (p = 0.007) times higher odds of attending TB services compared to those from control group.

### Cost assessment

We conducted a cost assessment to quantify the expenditures necessary to implement the active referral program (detailed data are presented in Table 2). We did not include the costs of tests performed at the substitution treatment center (these were performed solely for the research purposes and do not have to be administered routinely in this context) and costs related to TB screening in TB clinic. We used the mean hourly salary for medical nurses in Estonia. Thus for a substitution treatment center to organize one client's TB screening would cost an additional 18 EUR and for 200 clients 3,600 EUR.

**Table 2:** Costs related to referral of one patient

Task group	Resources used	Cost per unit	Units per patient	Price per patient
Counseling and referral	Nursing staff time	6.4 EUR per hour (including all taxes)	0.5 hour	3.2 EUR
Transportation	Substitution treatment center's vehicle	2L of gas + 1 hour of driving staff time = 2 EUR + 6.4 EUR	1	8.4 EUR
Incentives	Food voucher	6.4 EUR per voucher	1 voucher	6.4 EUR
Total				18 EUR

## Discussion

Drug users constitute a high-risk group for whom screening, prevention of infection, diagnosis, and treatment of TB may pose particular challenges. The development of TB services capable of engaging drug users (those both in and out of drug treatment programs) has potential for disrupting a significant chain of rapid TB transmission [1]. Drug treatment programs can provide a strategic setting for screening and directly observed preventive therapy, and for referring IDUs to needed health care, especially for TB and antiretroviral therapies [22,23].

None of our participants reported ever having had tuberculosis. At the same time 6.5% of all participants were positive for both HIV-antibodies and IGRA (as a marker of latent TB). These people constitute an especially high risk group for developing active tuberculosis. The risk of developing active TB could be as high as 5-10% per year [24].

Despite the fact that substitution treatment patients attend the clinic daily and many of them who are HIV-infected also attend infectious diseases clinics, only one third of them had had a chest X-ray performed within last 12 months prior to the recruitment. These results show the need to implement more active TB screening among methadone substitution treatment patients.

Results of our pilot program show that the uptake of TB screening services can be increased with more active referral, help in transportation and incentives. Other studies have also shown that monetary incentives can increase participation in programs (for example the return rate for TB skin test reading) [25].

Participants were very favorably inclined toward the pilot program and the high acceptance rate of the initial screening in substitution treatment center suggests that this type of approach can be incorporated into daily activities of substitution treatment program. Study staff did not meet any major obstacles and problems related to recruitment of participants, administration of the questionnaire and referrals.

Cost assessment showed that with relatively small additional investments the uptake of TB screening for IDUs can be considerably increased. In comparision - the mean cost of non-MDR-TB treatment case in Estonia is 3,251 EUR and of the MDR-TB patient - 8,469 EUR [26]. Early detection of TB improves the treatment outcomes [27] and reduces the period of infectiousness thus investment in TB screening for IDUs can be cost saving.

### Limitations

Small sample size, recruitment in only one centre, and sampling in methadone treatment program would not have accessed important groups of active injecting drug users, and therefore the results cannot be generalized to the whole IDU population. Generalisability might be further affected by the modest response rate among methadone center clients. Further, given that the HIV prevalence among methadone center clients was very high additional research is needed in terms on need and methods for screening among IDUs not in contact with harm reduction services. However, the main reason for declining study participation was recent contact with TB services and thus our intervention reached those in most need for TB screening.

## Conclusions

This pilot study illustrates the urgent need for scaling up TB screening among IDUs and the value of more active approach in referring substitution treatment patients to TB services.

## List of abbreviations used

CDC: Centers for Disease Control and Prevention; DOTS: directly observed treatment short course; EUR: euro; IDU: injecting drug user; IGRA: interferon gamma release assay; MDR: multi-drug resistant; TB: tuberculosis

## Competing interests

The full content of this paper has not been published elsewhere, nor is it being considered elsewhere, nor are there any conflicts of interest contained therein. Preliminary findings from this study have been presented at the XVII International AIDS Conference (Mexico City, Mexico, August 3-8, 2008). Abstract titled: Pilot program for tuberculosis control among methadone substitution treatment patients in Estonia (Rüütel K, Uusküla A, Loit HM).

## Authors' contributions

KR, AU, HML and KK designed the study. KR and TS supervised the data collection. KR, AU and LAM designed the data analysis and structure of the manuscript. KR conducted the statistical analysis. KR wrote the first draft of the manuscript. All of the authors contributed to the final version of the manuscript. All of the authors read and approved the final manuscript.
